# Supplementary material for: Spike in Asthma Healthcare Presentations in Eastern England during June 2021: A Retrospective Observational Study Using Syndromic Surveillance Data
Source: Int J Environ Res Public Health. 2021 Nov 24;18(23):12353. doi: 10.3390/ijerph182312353 (PMC8657080; doi:10.3390/ijerph182312353)
Supplement: Supplementary file 1 [file ijerph-18-12353-s001.zip › ijerph-1451507-supplementary.pdf]

Supplementary figures

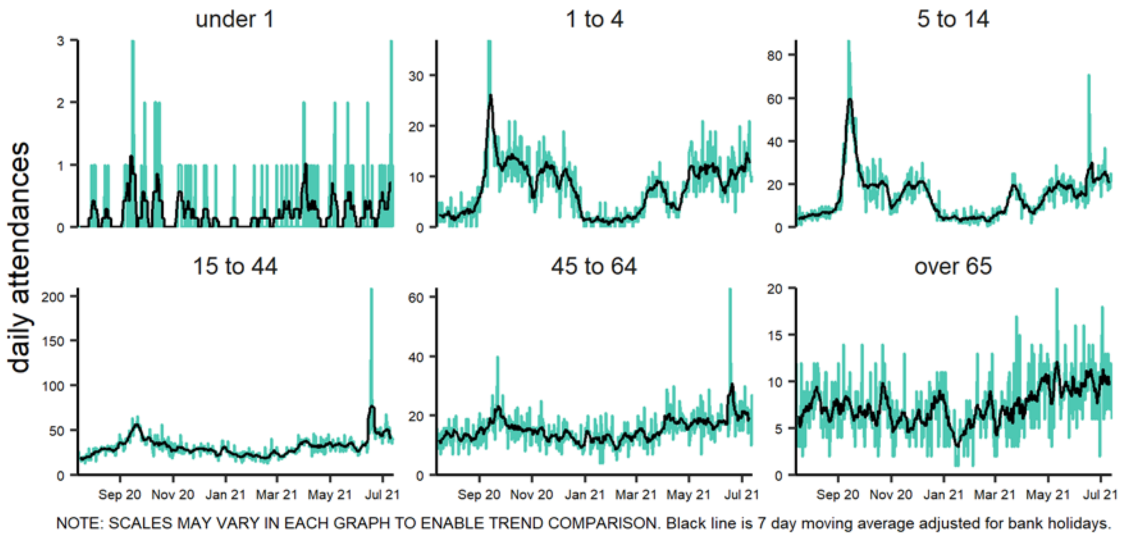

Figure S1. Emergency department asthma attendances (England) by age group (14/7/2020 – 13/7/2021).

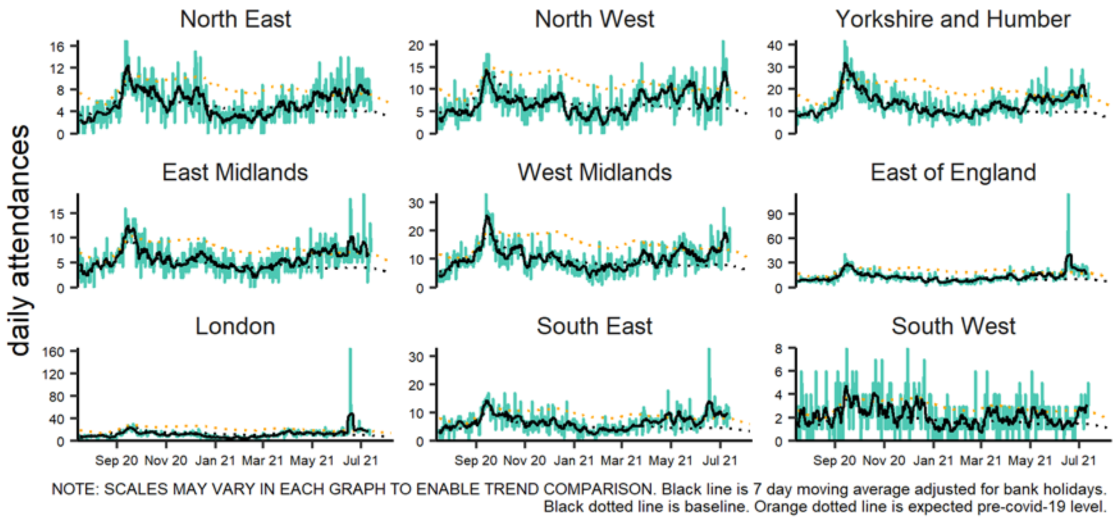

Figure S2. Emergency department asthma attendances (all ages) by region of England (14/7/2020 – 13/7/2021).

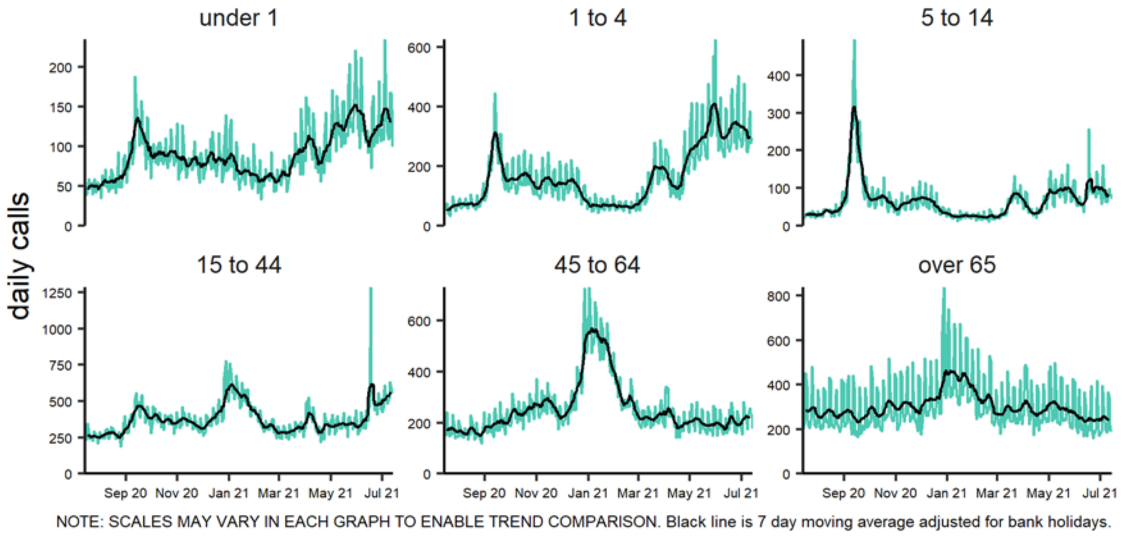

Figure S3. NHS 111 difficulty breathing calls (England) by age group (14/7/2020 – 13/7/2021).

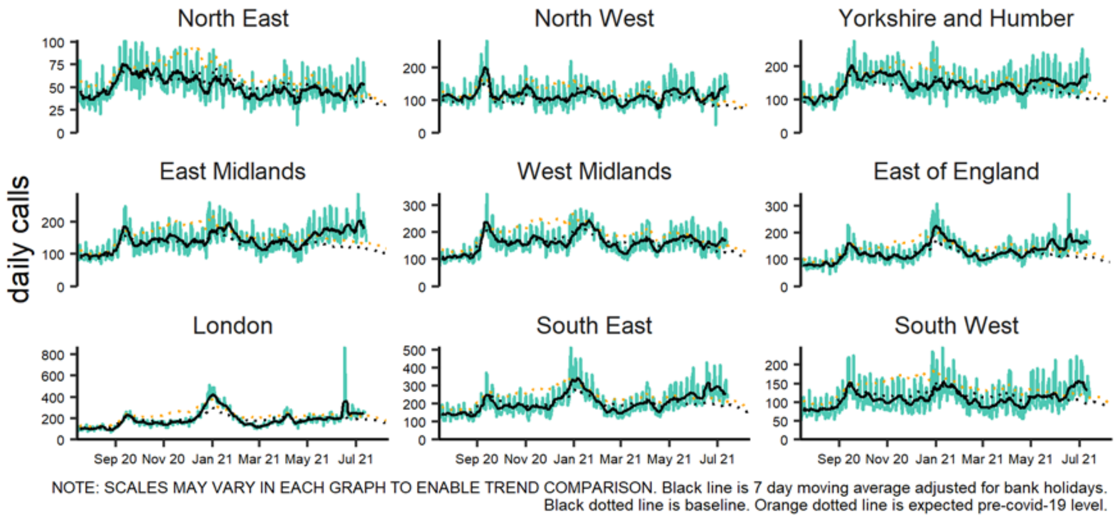

Figure S4. NHS 111 difficulty breathing calls (all ages) by region of England (14/7/2020 – 13/7/2021).

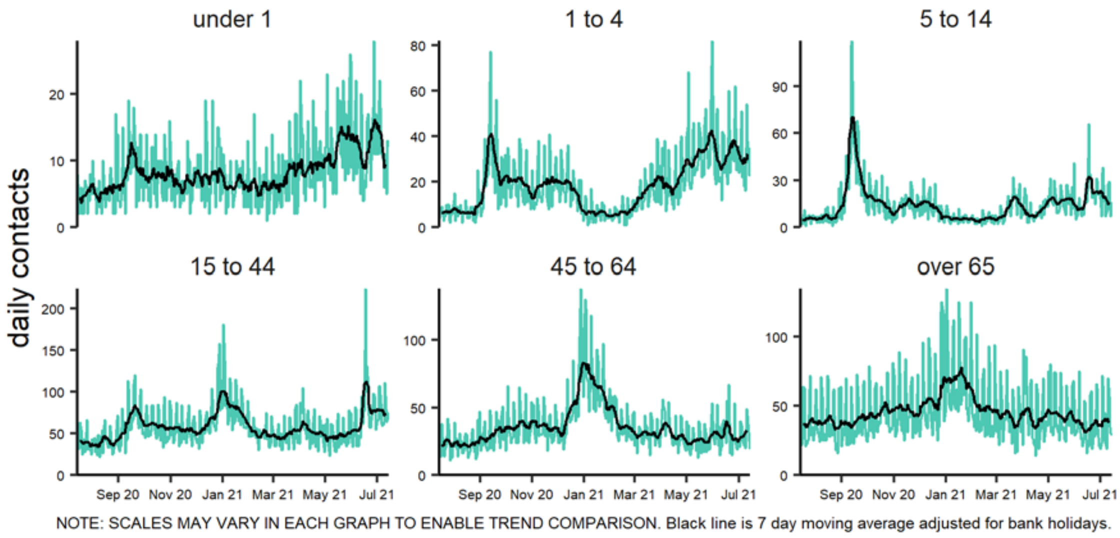

Figure S5. GP out of hours difficulty breathing/wheeze/asthma contacts (England) by age group (14/7/2020 – 13/7/2021).

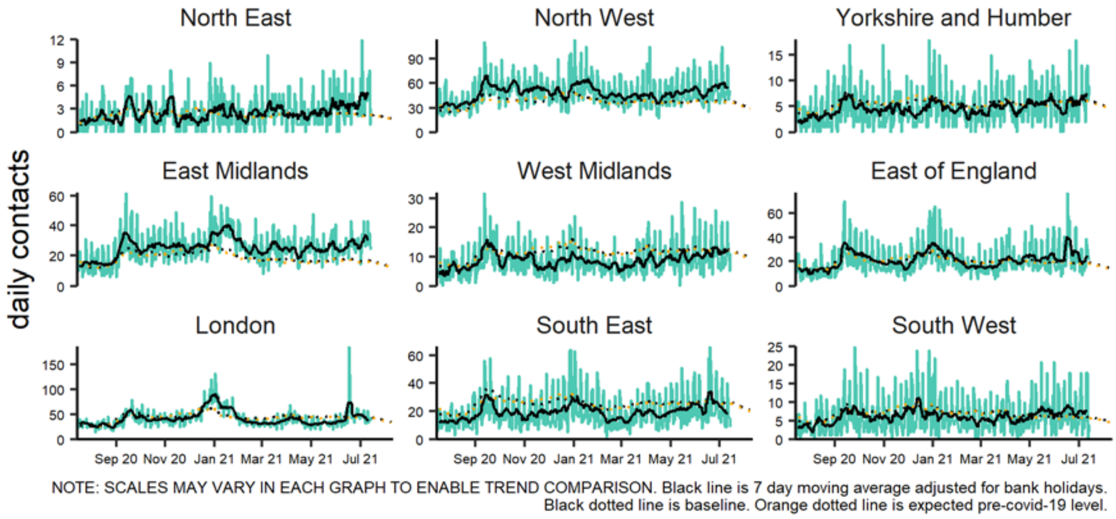

Figure S6. GP out of hours difficulty breathing/wheeze/asthma contacts (all ages) by region of England (14/7/2020 – 13/7/2021).

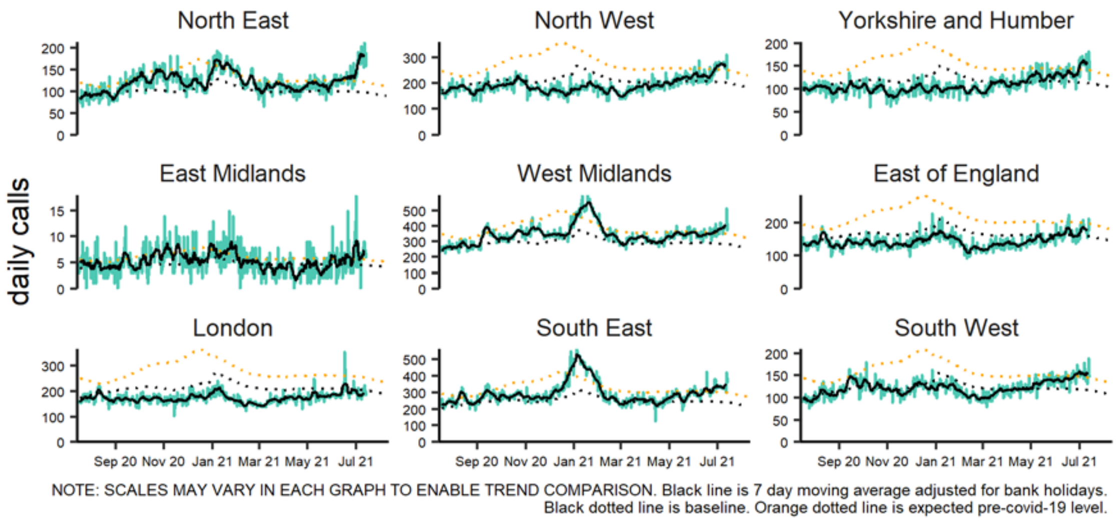

Figure S7. Ambulance breathing problems calls (all ages) by region of England (14/7/2020 – 13/7/2021).

Supplementary tables

Supplementary Table S1. Clinical coding systems and diagnostic codes mapped to the syndromic asthma and difficulty breathing indicators used in this study.

A. Emergency department: asthma

Codes ǀ Descriptor	Coding system
195967001 ǀ Asthma (disorder)	SnomedCT[1,2]
304527002 ǀ Acute Asthma	SnomedCT[1,2]
708038006 ǀ Acute exacerbation of asthma (disorder)	SnomedCT[1,2]
281239006 ǀ Exacerbation of asthma (disorder)	SnomedCT[1,2]
389145006 ǀ Allergic asthma	SnomedCT[1,2]
708090002 ǀ Acute severe exacerbation of asthma (disorder)	SnomedCT[1,2]
57546000 ǀ Asthma with status asthmaticus (disorder)	SnomedCT[1,2]
233678006 ǀ Childhood asthma (disorder)	SnomedCT[1,2]
445427006 ǀ Seasonal asthma (disorder)	SnomedCT[1,2]
394967008 ǀ Suspected asthma (situation)	SnomedCT[1,2]
708093000 ǀ Acute exacerbation of allergic asthma	SnomedCT[1,2]
1751000119100 ǀ Acute exacerbation of chronic obstructive airways disease with asthma (disorder)	SnomedCT1,[2]

B. General practitioner out of hours: difficulty breathing/wheeze/asthma

Codes ǀ Descriptor	Coding system
H333. ǀ Acute exacerbation of asthma	Read CTV3[3]
H33.. ǀ Asthma	Read CTV3[3]
H33z. ǀ Asthma unspecified	Read CTV3[3]
173.. ǀ Breathlessness	Read CTV3[3]
23B2. ǀ Bronchial breathing	Read CTV3[3]
1738. ǀ Difficulty breathing	Read CTV3[3]
2322. ǀ Dyspnoea	Read CTV3[3]
2326. ǀ Expiratory wheeze	Read CTV3[3]
232C. ǀ Noisy breathing	Read CTV3[3]
R061. ǀ Stridor	Read CTV3[3]
1737. ǀ Wheezing	Read CTV3[3]

C. NHS 111 calls and online assessments: difficulty breathing

Descriptor	Code system
Difficulty breathing pathway	NHS Pathways[4]

D. Ambulance: breathing problems

Descriptor	Code system
Breathing problems chief complaint	NHS Pathways
Breathing problems chief complaint	Advanced Medical Priority Dispatch System (AMDPS)[5]

[1] SNOMED CT 2012. International Health Terminology Standards Development Organisation. Available online: http://www.ihtsdo.org/snomed-ct/; [2] Emergency Care Dataset. NHS Digital. Available online: https://digital.nhs.uk/data-and-information/data-collections-and-data-sets/data-sets/emergency-care-data-set-ecds; [3] Read codes. NHS Digital. Available online: https://digital.nhs.uk/services/terminology-and-classifications/read-codes; [4] NHS Pathways. NHS Digital. Available online: https://digital.nhs.uk/services/nhs-pathways; [5] Ambulance Response Programme. University of Sheffield. Available online: https://www.england.nhs.uk/wp-content/uploads/2017/07/ARPReport_Final.pdf. all accessed on 23 November 2021.

Supplementary Table S2. Observed and expected cases of asthma and difficulty breathing across age groups 17 June 2021.

**Syndromic system**	**Indicator**	**<1**			**1-4**			**5-14**			**15-44**			**45-64**			**65+**		
		**Exp[1]**	**Obs[2]**	**% inc[3]**	**Exp**	**Obs**	**% inc**	**Exp**	**Obs**	**% inc**	**Exp**	**Obs**	**% inc**	**Exp**	**Obs**	**% inc**	**Exp**	**Obs**	**% inc**
Emergency department	Asthma	0	0	-100	13	19	43	19	76	292	47	165	248	21	61	187	13	13	2
NHS 111 calls	Difficulty breathing	124	103	-17	313	250	-20	69	256	270	349	1286	268	175	251	44	220	217	-1
NHS 111 online	Difficulty breathing	N/A[4]	N/A	N/A	N/A	N/A	N/A	N/A	N/A	N/A	N/A	N/A	N/A	N/A	N/A	N/A	N/A	N/A	N/A
GP out of hours	Difficulty breathing/ wheeze/ asthma	10	6	-41	29	22	-24	15	66	347	49	224	354	25	53	112	32	23	-28
Ambulance	Breathing problems	N/A	N/A	N/A	N/A	N/A	N/A	N/A	N/A	N/A	N/A	N/A	N/A	N/A	N/A	N/A	N/A	N/A	N/A

[1] 'expected' – number of daily counts expected on a weekday (Monday to Friday) based upon activity recorded in the preceding four weeks (weekdays inclusive through 24 May to 16 June 2021); [2] 'observed' – average number of daily counts observed based upon activity on 17 June 2021; [3] 'percentage increase' observed on 17 June 2021; [4] N/A – age specific data not available for the NHS 111 online and ambulance surveillance systems.
